# The effects of psilocybin and MDMA on between-network resting state functional connectivity in healthy volunteers

**DOI:** 10.3389/fnhum.2014.00204

**Published:** 2014-05-27

**Authors:** Leor Roseman, Robert Leech, Amanda Feilding, David J. Nutt, Robin L. Carhart-Harris

**Affiliations:** ^1^Centre for Neuropsychopharmacology, Division of Brain Sciences, Department of Medicine, Imperial College LondonLondon, UK; ^2^Computational, Cognitive and Clinical Neuroscience Laboratory, Division of Brain Sciences, Department of Medicine, Imperial College LondonLondon, UK; ^3^The Beckley FoundationOxford, UK

**Keywords:** psilocybin, MDMA, serotonin, 5HT2A, resting state, functional connectivity, brain networks, psychedelic

## Abstract

Perturbing a system and observing the consequences is a classic scientific strategy for understanding a phenomenon. Psychedelic drugs perturb consciousness in a marked and novel way and thus are powerful tools for studying its mechanisms. In the present analysis, we measured changes in resting-state functional connectivity (RSFC) between a standard template of different independent components analysis (ICA)-derived resting state networks (RSNs) under the influence of two different psychoactive drugs, the stimulant/psychedelic hybrid, MDMA, and the classic psychedelic, psilocybin. Both were given in placebo-controlled designs and produced marked subjective effects, although reports of more profound changes in consciousness were given after psilocybin. Between-network RSFC was generally increased under psilocybin, implying that networks become less differentiated from each other in the psychedelic state. Decreased RSFC between visual and sensorimotor RSNs was also observed. MDMA had a notably less marked effect on between-network RSFC, implying that the extensive changes observed under psilocybin may be exclusive to classic psychedelic drugs and related to their especially profound effects on consciousness. The novel analytical approach applied here may be applied to other altered states of consciousness to improve our characterization of different conscious states and ultimately advance our understanding of the brain mechanisms underlying them.

## Introduction

Psychedelic drugs have been used throughout history by different cultures as a means of altering consciousness. They are powerful tools for understanding the neurobiology of consciousness yet they have been underutilized by modern science, arguably due to political rather than scientific circumstances (Nutt et al., 2013). The majority of consciousness research has focused on states of reduced consciousness such as coma and sleep (Laureys, 2005). Indeed, consciousness has been defined as that which is lost during dreamless sleep (Tononi, 2004) but consciousness can also be studied in terms of changes in the mode or style of waking consciousness, such as is seen in the psychedelic state. Another popular model of consciousness describes it using two parameters: (1) wakefulness or arousal and (2) awareness (Laureys et al., 2009). It is recognized that these parameters have a mostly linear relationship; however, REM sleep and the vegetative state are considered anomalies, since the former involves greater awareness than would be predicted by wakefulness and the latter displays less (Laureys et al., 2009). The position of the psychedelic state in this model has never been considered before and it presents another interesting anomaly. There is no evidence of reduced wakefulness in the psychedelic state and although awareness is altered, it would be misleading to say that it is reduced. Indeed, the psychedelic state has been referred to as an “expansive” state of consciousness (Huxley, 1959). Thus, it is important to investigate what the neurobiological basis of this putative broadening of consciousness is.

One of the most popular theories of consciousness is the “information integration” theory of Tononi (2012). This proposes that consciousness depends on the presence of two key parameters: (1) information and (2) integration. *Information* is derived from information theory (Shannon and Weaver, 1949) and in the context of consciousness, refers to the potential size of the repertoire of different metastable states (Tognoli and Kelso, 2014) (or “sub-states”) the mind/brain can enter over time. *Integration* refers to the capacity of the mind/brain to integrate processes into a collective whole. The parameter of *awareness* is likely to be related to the property of *information*, since the greater the repertoire of sub-states the mind can enter, and the easier it can move between these, the broader consciousness will be.

In recent years, there has been an increasing interest in human fMRI measures of resting state functional connectivity (RSFC) (Damoiseaux and Greicius, 2009). Resting state networks (RSN) can be identified using seed-based approaches (Biswal et al., 1995) and independent component analysis (ICA) (Beckmann et al., 2005). These RSNs resemble stimulus-evoked networks (Smith et al., 2009) and may be thought of as metastable sub-states making-up a particular (macro) state of consciousness (Carhart-Harris et al., 2014a). Thus, one way to describe the quality of a macro-state of consciousness may be to investigate the integrity and dynamics of its sub-states and how they interact with each other. One way this can be done is by looking at the internal stability (integrity) of an RSN, i.e., reflected in the strength of the coupling between its constituent nodes. For example, we have found decreased intra-RSN connectivity post-psilocybin with both fMRI (Carhart-Harris et al., 2012a) and magnetoencephalography (MEG) (Muthukumaraswamy et al., 2013), implying a general breakdown of the integrity or internal stability of RSNs under psilocybin.

Another way to address the behavior of a system's sub-states is to look at their relationship with each other, e.g., by measuring between-RSN functional connectivity or coupling. A frequently investigated RSN is the default mode network (DMN) (Raichle et al., 2001). The DMN is known to be more active during rest than during goal-directed cognition and its activity has been found to be “anti-correlated” or at least uncorrelated or orthogonal with activity in networks that are engaged during goal-directed cognition - referred to generically as “task positive networks” or TPNs. This anticorrelation is preserved under task free conditions (Fox et al., 2005), implying that it is an important feature of normal consciousness, perhaps accounting for the distinction between externally focused cognition and introspection (Carhart-Harris et al., 2012b). We recently found that the classic psychedelic drug psilocybin reduces the anticorrelation between DMN and a number of TPNs during resting conditions, and this was interpreted as a decrease in the natural distinction between externally-focused attention and introspection (Carhart-Harris et al., 2012b), which is relevant to the notion of “ego-boundaries,” i.e., an agent's sense of being apart from or separate to its environment. It would be a natural extension of the above analysis to address the full gamut of between-RSN FC identified by ICA rather than just focusing on just the DMN-RSN RSFC. This was the aim of the present study.

The primary focus of the present paper is the classic psychedelic state and determining its underlying neurodynamics as measured with fMRI. However, in order to understand the psychedelic state, it is useful to compare it with other states of consciousness to see how it relates to these. Thus, the present analysis focuses on the brain effects of a classic psychedelic drug, psilocybin (the active component of magic mushrooms) and compares this with the effects of the pro-serotonergic stimulant, 3–4 methylenedioxymethamphetanine, MDMA. MDMA is a potent monoamine releaser that produces an acute euphoria in most individuals but it is not considered a classic psychedelic, as psilocybin is. Direct 5-HT_2A_R stimulation is the defining pharmacological property of classic serotonergic psychedelics, but relative to classic psychedelics, MDMA has a far weaker affinity for the 5-HT_2A_ receptor (Green et al., 2003). Instead, MDMA produces a more generalized, non-selective activation of monoamine receptors by increasing the concentration of their endogenous ligands in the synapse via transporter-mediated release (Green et al., 2003). The primary subjective effects of MDMA include increased positive mood, heightened sensations and prosocial sentiments and although it can produce mild visual hallucinatory phenomena, it does not alter consciousness in the same fundamental manner as classic psychedelics (Gouzoulis-Mayfrank et al., 1996).

Thus, comparing changes in RSFC under psilocybin and MDMA can enable us to isolate and identify effects that are unique to the psychedelic-induced altered state of consciousness produced by classic psychedelics such as psilocybin. Considering the previous findings of decreased intra-RSN FC and DMN-TPN anti-correlation under psilocybin (Carhart-Harris et al., 2012a,b; Muthukumaraswamy et al., 2013), we hypothesized that the normal differentiation between RSNs would be affected by psilocybin such that RSNs whose activity is usually highly correlated would show reduced RSFC under psilocybin (but not MDMA) and that networks that are normally anti-correlated would show reduced anti-correlation under psilocybin (but not MDMA). If the hypothesized effects are present under psilocybin but absent under MDMA, this will strengthen the inference that they are specifically related to psilocybin more profound effects on consciousness.

## Materials and methods

### Design

#### Psilocybin

This is an entirely new analysis on a previously published data set (Carhart-Harris et al., 2012a,b). This was a within-subjects placebo-controlled study that was approved by a local NHS Research Ethics Committee and Research and Development department, and conducted in accordance with Good Clinical Practice guidelines. A Home Office License was obtained for storage and handling of a Schedule 1 drug. The University of Bristol sponsored the research. The research was carried out at CUBRIC, University of Cardiff.

#### MDMA

This is also an entirely new analysis on a previously published dataset (Carhart-Harris et al., 2014b). This was a within-subjects, double-blind, randomized, placebo-controlled study. Participants were scanned twice, 7 days apart, once after MDMA and once after placebo. The study was approved by NRES West London Research Ethics Committee, Imperial College London's Joint Compliance and Research Office (JCRO), Imperial College's Research Ethics Committee (ICREC), the Head of Imperial College's Department of Medicine, Imanova Center for Imaging Science and Imperial College London's Faculty of Medicine, and was conducted in accordance with Good Clinical Practice guidelines. A Home Office License was obtained for the storage and handling of a Schedule 1 drug and Imperial College London sponsored the research.

### Participants

#### Psilocybin

Fifteen healthy subjects took part: 13 males and 2 females (mean age = 32, *SD* = 8.9). Recruitment was via word of mouth. All subjects were required to give informed consent and undergo health screens prior to enrolment. Entry criteria were: at least 21 years of age, no personal or immediate family history of a major psychiatric disorder, substance dependence, cardiovascular disease, and no history of a significant adverse response to a hallucinogenic drug. All of the subjects had used psilocybin at least once before (mean number of uses per subject = 16.4, *SD* = 27.2) but not within 6 weeks of the study.

#### MDMA

The original study sample comprised of 25 healthy participants (mean age = 34, *SD* = 11, 7 females) with at least 1 previous experience with MDMA. None of the participants had used MDMA for at least 7 days and other drugs for at least 48 h, and this was confirmed by a urine screen. As a conservative step to control for between-study differences in the global intensity of the subjective effects produced by the different drugs, 11 subjects who gave ratings of <50% for the intensity of MDMA's effects were excluded from the analysis. This step meant that ratings of drug effects intensity were comparable across the two studies (i.e., the mean intensity of psilocybin's subjective effects was 67 ± 19 at peak and MDMA's was 69 ± 15). An additional subject was excluded because of significant head movements (mean head motion > one voxel width). Thus, a total of 13 subjects were included in the analysis (i.e., 12 excluded). An alcohol Breathalyzer test confirmed that none of the participants had recently consumed alcohol. For the sample of 13, participants had used MDMA an average of 29 (±35) times before (range = 1–100) and the mean time since last use was 983 (±1998) days (range = 7–6570 days). Participants were screened for general health, MR-compatibility and present mental health. Screening involved routine blood tests, electrocardiogram, heart rate, blood pressure and a brief neurological exam. All subjects were deemed physically and mentally healthy at the time of study entry and none had any history of drug or alcohol dependence.

### Anatomical scans

#### Psilocybin

Imaging was performed on a 3T GE HDx system. Anatomical scans were performed before each functional scan. These were 3D fast spoiled gradient echo scans in an axial orientation, (1 mm isotropic voxels).

#### MDMA

Imaging was performed on a 3T Siemens Tim Trio (Siemens Healthcare, Erlangen, Germany) using a 32-channel phased array head coil. Anatomical reference images were acquired using the ADNI-GO recommended MPRAGE parameters (1 mm isotropic voxels).

### Drug and scanning parameters

#### Psilocybin

All subjects underwent two 12-min eyes-closed resting-state blood oxygen–level dependent (BOLD) fMRI scans on 2 separate occasions at least 7 days apart: placebo (10 ml saline, 60-s intravenous injection) was given on 1 occasion and psilocybin (2 mg dissolved in 10 ml saline) on the other. Seven of the subjects received psilocybin in scan 1, and 8 received it in scan 2. Injections were given manually by a study doctor situated within the scanning suite. The 60-s infusions began exactly 6 min after the start of the 12-min scans. Subjective ratings were given post-scan using visual analog scales (VAS). The subjective effects of psilocybin were felt almost immediately after injection and were sustained for the duration of the scan.

#### MDMA

Two BOLD resting-state scans were performed during each functional scanning session (duration of functioning scanning = 60 min). The first resting-state BOLD scan took place 60 min after capsule ingestion and the second resting-state BOLD scan occurred 113 min after capsule ingestion. Peak subjective effects were reported 100 min post administration of MDMA, generally consistent with the plasma t-max of MDMA (Kolbrich et al., 2008). The order of MDMA and placebo administration was counterbalanced.

### fMRI data acquisition

#### Psilocybin

BOLD-weighted fMRI data were acquired using a gradient echo planar imaging sequence, 3 mm isotropic voxels, *TR* = 3000 ms, *TE* = 35 ms, field-of-view = 192 mm, 90° flip angle, 53 axial slices in each TR, parallel acceleration factor = 2, 64 × 64 acquisition matrix. The psilocybin and placebo scans for this analysis were of 5 min (1 min post infusion).

#### MDMA

BOLD-weighted fMRI data were acquired using a gradient echo planar imaging sequence, 3 mm isotropic voxels, *TR* = 2000 ms, *TE* = 31 ms, field-of-view = 192 mm, 80° flip angle, 36 axial slices in each TR, GRAPPA acceleration = 2, bandwidth = 2298 Hz/pixel. For each condition, MDMA and placebo, two scans were used for the analysis, each one of 6 min (performed 60 min and 113 min post-capsule ingestion)

### Resting state networks (RSN)

**Figure 1:**
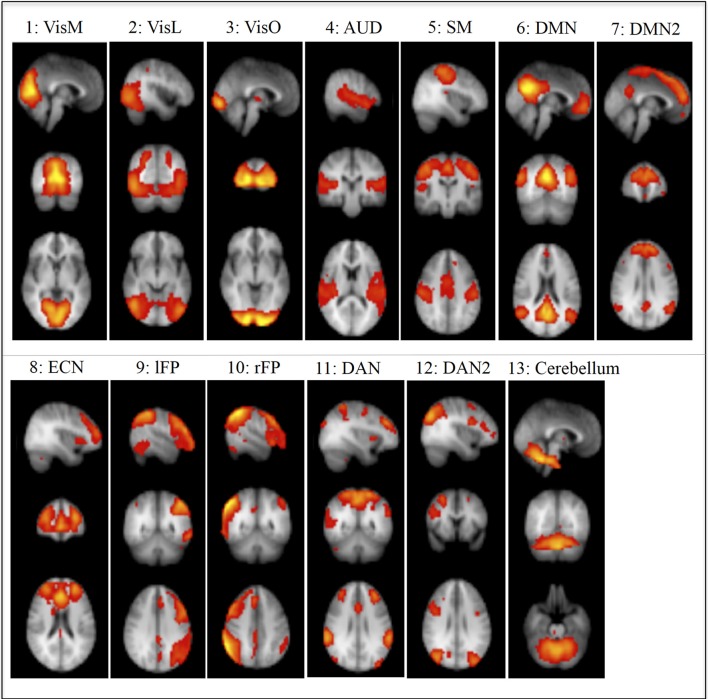
**Non-noise resting State Networks (RSN) from Smith et al., 2009:** (1) Visual–Medial (VisM), (2) Visual–Lateral (VisL), (3) Visual–Occipital pole (VisO), (4) Auditory (AUD), (5) Sensorimotor (SM), (6) Default Mode Network (DMN), (7) DMN2–A hybrid of anterior DMN and Executive Control Network, (8) Executive Control Network (ECN), (9) left Frontoparietal Network (lFP), (10) right Frontoparietal Network (rFP), (11) Dorsal Attention Network (DAN), (12) DAN2, (13) Cerebellum. Ten of these components were given functional labels based on their correspondence to the BrainMap database of functional imaging studies. (RSNs 1, 2, 3, 4, 5, 6, 8, 9, 10, 13), additional networks (7, 11, 12) were labeled by the experimenters in the current study based on the regional distribution of activity.

We used RSNs that were identified in Smith et al. (2009) using ICA (Figure 1). Ten of these components were given functional labels based on their correspondence to the BrainMap database of functional imaging studies, involving task-evoked FMRI data from nearly 30,000 human subjects. These networks were: Visual-Medial Network (VisM), Visual-Lateral Network (VisL), Visual-Occipital pole Network (VisO), Auditory Network (AUD), Sensorimotor Network (SM), Default Mode Network (DMN), Executive Control Network (ECN), Left frontoparietal Network(lFP), Right frontoparietal Network (rFP) and Cerebellar network. In addition, we used three more components from Smith et al, that we named DMN2 (an anterior DMN and ECN hybrid), Dorsal Attention Network 1 and 2 (DAN1 and DAN2). Another 6 components were identified as non-neural noise (likely generated by head motion and non-neural physiological fluctuations).

### Preprocessing

All analyses were performed using the Functional Magnetic Resonance Imaging of the Brain (FMRIB) Software Library (FSL, www.fmrib.ox.ac.uk/fsl) (Smith et al., 2004). We used the standard imaging preprocessing FSL pipeline that involved brain extraction (Smith, 2002), motion correction using MCFLIRT (Jenkinson et al., 2002), spatial smoothing (FWHM) of 5 mm (Smith and Brady, 1997) and a high-pass filter of 100 s. The scans were registered to the subjects' T1-weighted high-resolution (2 × 2 × 2 mm) anatomical scans and were then registered to the Montreal Neurological Institute standard brain (2 × 2 × 2 mm) (Jenkinson et al., 2002). The data was resampled into 4 mm space as part of the default processing pipeline for Melodic and was done to make the analysis more computational efficient.

### Between networks functional connectivity (FC)

#### Psilocybin

**Figure 2:**
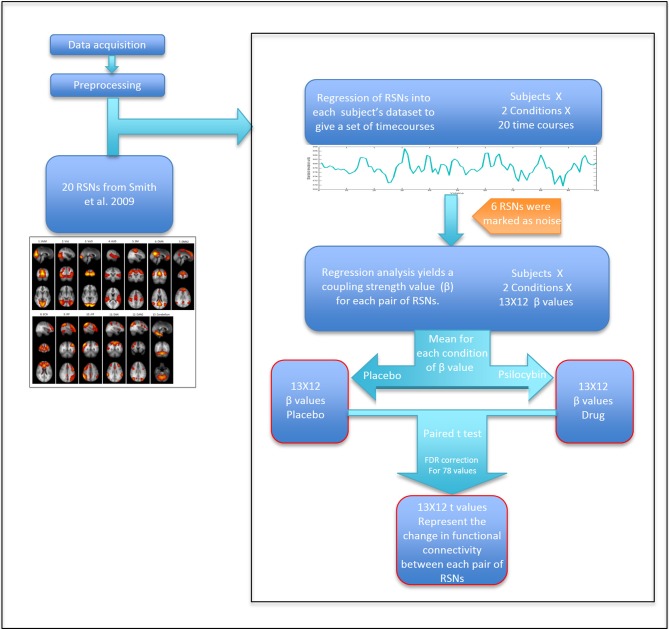
**Scheme of the analysis by steps**. Calculating *t*-values for each RSN pair that represent the change in coupling strength between placebo and drug.

To extract time courses for each subject for each RSN and for each condition, we back-projected the components from Smith et al. (2009) into each 4D fMRI dataset using a general linear model. Specifically, we took the 20 components ICA map from Smith et al. as the set of template ICAs for the dual regression pipeline. The first step of the “dual regression” pipeline was then applied to each 4D dataset, resulting in a specific timecourse for each component for each dataset (Beckmann et al., 2009). Between-RSN coupling was presented graphically using a 13 × 13 correlation (or more strictly, regression) matrix in which the color in each square represents a beta weight or coupling strength for the corresponding RSN-RSN pair. Specifically, these weights were calculated by entering the time course for a specific RSN as a dependent variable in a general linear model, with the time course of another RSN entered as an independent variable—with this procedure repeated for each RSN pair. The mean head motion under psilocybin and its placebo condition were 0.1 ± 0.05 mm and 0.06 ± 0.015 mm, respectively (*p* < 0.01). Therefore, to further partial out non-neural noise confounds, six motion time courses (estimated from the motion correction) and motion outliers (estimated using the “fsl_motionoutlier” command implemented in FSL), as well as the time courses for 6 non-neural noise components were entered as confounds (some of this noise is driven by head motion). The model resulted in a parameter estimate or unstandardized beta weight (β) representing the strength of functional coupling between each RSN pair. The general linear model was estimated twice for each RSN pair: with each RSN as dependent variable in one model and as an independent variable in the second model. Since we were not looking at effective or directed connectivity (Friston et al., 2003), we created a symmetrical connectivity matrix by averaging together each subject's two β values for each RSN pair. For each RSN pair, three results were calculated: (a) group mean β value for the placebo condition; (b) group mean β value for the psilocybin condition; (c) Paired *t*-test (2-tail) for the difference between the mean β values of each condition (Figure 2). To correct for multiple comparisons, a false discovery rate (FDR) threshold was calculated using *q* = 0.05 and *q* = 0.1 (*N* = 78).

#### MDMA

The MDMA RSFC was analyzed using the same procedure described above (Figure 2). The only difference was that there were two resting state scans in the MDMA study, so β values from the two scans (performed 60 min and 113 min post-capsule ingestion) were averaged together before comparing between the placebo and drug conditions. The mean head motion under MDMA and its placebo condition were 0.083 ± 0.036 mm and 0.061 ± 0.019 mm, respectively (*p* = 0.047). The same procedure to control for motion in the psilocybin analysis was used for MDMA.

## Results

### Subjective effects

#### Psilocybin

The subjective effects of psilocybin have been documented elsewhere (Carhart-Harris et al., 2011, 2012a). Briefly, the subjective effects of 2 mg psilocybin given as an intravenous injection over 60 s begin at the end of the injection period, reach a sustained peak after approximately 5 min, and subside completely after 45–60 min. Primary subjective effects include altered visual perception (e.g., hallucinated motion and geometric patterns), an altered sense of space and time, and vivified imagination. The intensity of psilocybin's global subjective effects was rated using a VAS format. The mean intensity at peak effects (5 min post-infusion) was 67% ±19.

#### MDMA

The subjective effects of MDMA are reported in a separate paper (Carhart-Harris et al., 2014b). At their peak, the average intensity of MDMA's global subjective effects was 69% ±15 (*n* = 13). There was no significant difference between intensity ratings under the two different drugs.

### Between networks FC

#### Psilocybin

**Figure 3:**
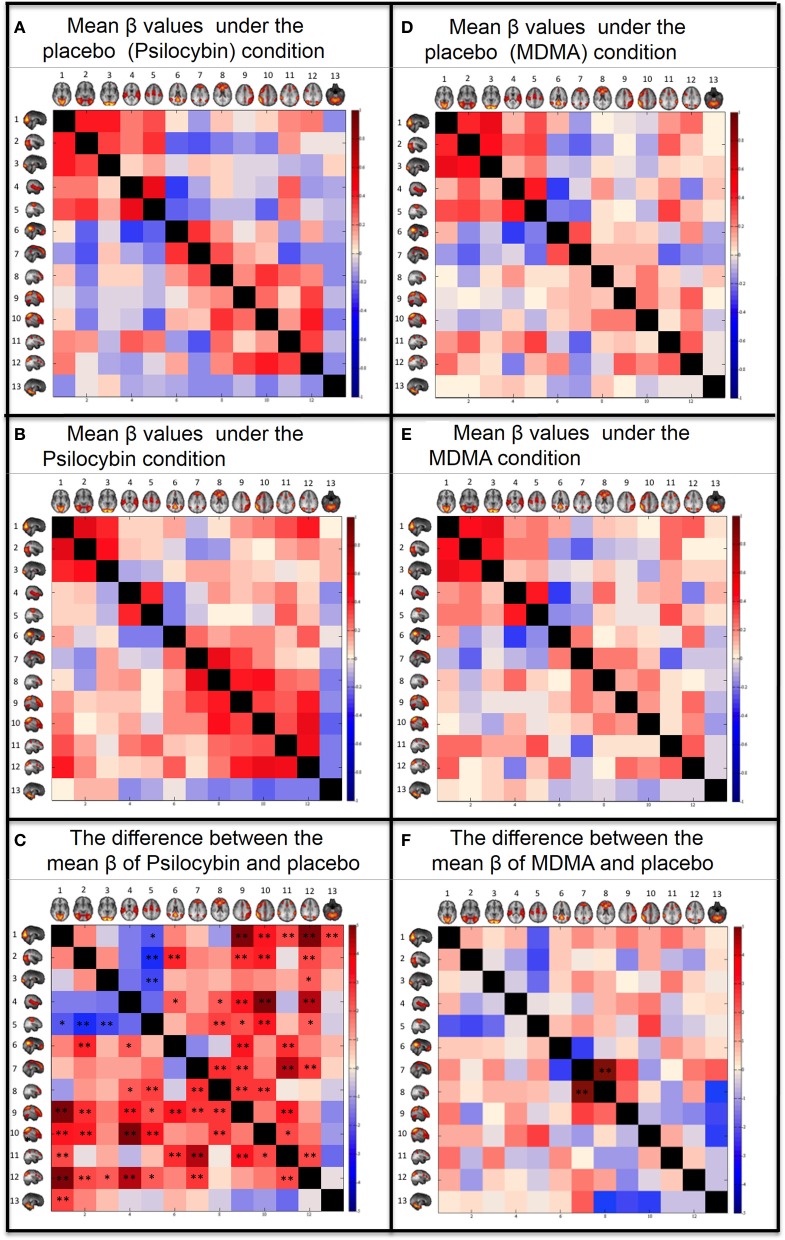
**Between networks resting state functional connectivity results**. Within each matrix, each colored square represents coupling between corresponding RSN pairs with the color of the square denoting the coupling strength **(A,B,D,E)** or change in coupling strength **(C,F)** between the RSN pairs (blue, negative coupling or a decrease in coupling; red, positive coupling or an increase in coupling). The six images are: **(A)** Group mean of β values for the placebo of psilocybin condition. **(B)** Group mean of β values for the psilocybin condition. **(C)** Paired *t*-test (2-tail) for the difference between the mean β values of psilocybin and placebo. **(D)** Group mean of β values for the placebo of MDMA condition. **(E)** Group mean of β values for the MDMA condition. **(F)** Paired *t*-test (2-tail) for the difference between the mean β values of MDMA and placebo. The networks from Smith et al., (2009) are: (1) Visual—Medial (VisM), (2) Visual—Lateral (VisL), (3) Visual—Occipital pole (VisO), (4) Auditory (AUD), (5) Sensorimotor (SM), (6) Default Mode Network (DMN), (7) DMN2—A hybrid of anterior DMN and Executive Control Network, (8) Executive Control Network (ECN), (9) left Frontoparietal Network (lFP), (10) right Frontoparietal Network (rFP), (11) Dorsal Attention Network (DAN), (12) DAN2, (13) Cerebellum. FDR correction for multiple comparison (*N* = *78*) was applied on the *t*-tests: *0.05 < *q* < 0.1. **q < 0.05.

The coupling strengths (β) for each condition can be seen graphically in the correlation matrixes in Figure 3 and numerically in the Supplementary material. For the placebo condition, see Figure 3A and Supplementary Table 1A and for the psilocybin condition see Figure 3B and Supplementary Table 1B. A paired *t*-test (2-tail) was done across subjects to compare the β values for each RSN pair in the drug and placebo (Figure 3C and Supplementary Table 1C). The results were corrected for multiple comparisons using FDR with *q* = 0.05 (resulting in a threshold of *p* < 0.0167) and *q* = 0.1 (resulting a threshold of *p* < 0.042). The RSN pairs that showed a significant decrease in coupling under psilocybin were: SM-VisM (*p* = 0.0265), SM-VisL (*p* = 0.0051) and SM-VisO (*p* = 0.0151). The RSN pairs that showed a significant increase in coupling were: VisM-lFP (*p* = 0.0001), VisM-DAN (*p* = 0.0156), VisM-rFP (*p* = 0.0023), VisM-DAN2 (*p* = 0.0002), VisM-Cerebellum (*p* = 0.0108), VisL-DMN (*p* = 0.0046), VisL-lFP (*p* = 0.0056), VisL-rFP (*p* = 0.0031), VisL-DAN2 (*p* = 0.0142), VisO-DAN2 (*p* = 0.0256), AUD-DMN (*p* = 0.028), AUD-ECN (*p* = 0.0323), AUD-lFP (*p* = 0.0029), AUD-rFP (*p* = 0.0001), AUD-DAN2 (*p* = 0.0005), SM-ECN (*p* = 0.0105), SM-lFP (*p* = 0.022), SM-rFP (*p* = 0.0026), SM-DAN2 (*p* = 0.034), DMN-lFP (*p* = 0.0029), DMN-DAN (*p* = 0.0058), DMN2-ECN (*p* = 0.0071) DMN2-lFP (*p* = 0.0101), DMN2-DAN (*p* = 0.0005), DMN2-DAN2 (*p* = 0.0091), ECN-lFP (*p* = 0.0077), ECN-rFP (*p* = 0.0098), lFP-DAN (*p* = 0.0026), rFP-DAN (*p* = 0.0187), and DAN-DAN2 (*p* = 0.0161).

#### MDMA

The same analysis as above was repeated for the MDMA condition using a *q* of 0.05, resulting in a threshold of *p* < 0.0006 and *q* = 0.1, resulting in a threshold of *p* < 0.0012. Only one RSN pair showed a significant change in coupling under MDMA, i.e., increased coupling between the DMN2-ECN (*p* = 0.0001).

### Differences in movement

Both drugs showed significant, yet relatively modest, increased head motion between conditions. The mean head motion under psilocybin and its placebo condition were 0.1 ± 0.05 mm and 0.06 ± 0.015 mm, respectively (*p* < 0.01). The mean head motion under MDMA and its placebo condition were 0.083 ± 0.036 mm and 0.061 ± 0.019 mm, respectively (*p* = 0.047). Power et al. (2012) suggest that head motion can change the results of RSFC, therefore, in the regression analysis, we added several motion confounds: six motion time courses, motion outliers [similar to the procedure of scrubbing within regression (spike regression) mentioned by Yan et al. (2013) and Satterthwaite et al. (2013)] and time courses of RSNs that were driven by motion. However, it still remains possible that the increased movement under the drugs may have caused the changes in RSFC. Hence, we investigated if there was a relationship between the change in estimated motion (mean framewise displacement) between placebo and drug and the change in coupling strength (for pairs of RSNs that showed significant differences in coupling). For most of the RSN pairs no relationship was found (*p* < 0.05). However, under psilocybin, there were significant correlations with motion in the following RSN pairs: VisM-SM (*p* = 0.002), VisL-SM (*p* = 0.001), VisO-SM (*p* = 0.02), VisL-DMN (*p* = 0.03), VisM-rFN (*p* = 0.048), VisL-rFN (*p* = 0.01), VisO-DAN2, DMN-lFN (*p* = 0.001), DMN-DAN (*p* = 0.01). For that reason, the significant results of these RSN pairs should be approached with caution.

## Discussion

To our knowledge, this is the first analysis to test the effects of different pharmacological agents using a standard ICA-derived template of RSNs to construct between-network functional connectivity matrixes for different drug states. This approach may have wider application, enabling researchers to determine connectivity “fingerprints” for characterizing different states of consciousness, i.e., not only those induced by pharmacological agents but sleep states and even pathological states. This will enable informed comparisons to be made between different states, potentially allowing us to categorize different states based on their connectivity profiles. Functional connectivity matrixes have been used before to differentiate between pathology states such as schizophrenia and bipolar disorder (Mamah et al., 2013) and here we suggest that they could be used more broadly to characterize states of consciousness, including those induced by psychoactive drugs.

Probably the most striking result of the present study was the marked increases in between-network RSFC under psilocybin. These increases were evident for heteromodal networks, both in terms of increased unimodal-heteromodal (e.g., AUD-rFP) and heteromodal-heteromodal network RSFC (e.g., lFP-ECN). Based on previous analyses (Carhart-Harris et al., 2012b), we had predicted that RSN pairs with weak or negative RSFC at baseline would show increased coupling post-psilocybin, and this was found (e.g., DMN-VisL). However, the increases in between-network RSFC were more fundamental than this, being evident for RSN pairs that were already positively coupled at baseline (e.g., DMN2-ECN). The increase in correlated brain activity across normally distinct brain networks was particularly true for heteromodal RSNs, where the distribution of 5-HT_2A_ receptors is known to be highest (Erritzoe et al., 2010) and 5-HT_2A_ receptor stimulation is linked to desynchronous cortical activity (Riba et al., 2002; Wood et al., 2012; Muthukumaraswamy et al., 2013) and network disintegration (Muthukumaraswamy et al., 2013; Carhart-Harris et al., 2014a).

The pattern of increased between-network RSFC under psilocybin did not apply universally for the whole of the brain. Decreased RSFC was observed between the three visual RSNs and the sensorimotor network [these networks are known to be highly connected (Wise et al., 1997; Van Den Heuvel et al., 2008)], and there was a general trend toward decreased unimodal-unimodal network RSFC (e.g., VisM-AUD and SM-AUD showed decreased RSFC under psilocybin but this failed to survive FDR correction, see Supplementary Table 1). However these decreases can also be explained by the changes in head motion between conditions and further work is required to test whether these decreases in sensory RSN RSFC under psilocybin relate to the drug's characteristic perceptual/hallucinatory effects.

Previous neuroimaging studies with psychedelics have so far failed to reveal a simple and compelling explanation for their characteristic hallucinogenic effects (Vollenweider et al., 1997; Carhart-Harris et al., 2012a; Muthukumaraswamy et al., 2013) (but see De Araujo et al., 2012) and so drug-induced visual hallucinations remain poorly understood. Under normal conditions, activity in the visual cortex is driven by and thus anchored to visual input. Moreover, activity in other networks (e.g., the DMN), concerned with other distinct functions (e.g., introspection), is often weakly or inversely coupled to visual activity (e.g., see the pale and blue colored squares for the visual-RSN pairs in Figures 3A,D). Thus, increased communication between the visual system and systems that are usually reserved for distinct functions may lead to erroneous perceptual associations. For example, increased DMN-visual network RSFC, may relate to an increased influence of imagination (mediated by the DMN) on visual perception (mediated by the visual networks). A similar process may occur in situations of sensory deprivation where sensory processing becomes decoupled from sensory stimulation, allowing the system to “free-wheel” with the potential for the spontaneous emergence of internally-generated percepts. Decreased cross-modality RSFC and increased unimodal to heteromodal network RSFC may be a common characteristic of such states but future studies are required to test this. For example, comparisons between the present results and changes in RSFC in the meditative state could inform these speculations.

Given reports of synesthesia-like experiences under psychedelics (e.g., participants reported that the noise of the MR scanner influenced the rate and content of their closed eye visual hallucinations Carhart-Harris et al., 2012a and see also Luke and Terhune, 2013) one may have predicted increased cross-modality communication under psilocybin rather than the decreased coupling that was observed here. However, it has yet to be determined whether synesthesia-like experiences in drug-induced altered states of consciousness are qualitatively and mechanistically related to synesthesia experienced outside of this context and it is also worth noting that increased visual to heteromodal cortical functional connectivity has been found in color-grapheme synesthesia (Dovern et al., 2012; Sinke et al., 2012) as well as in the present study.

Taking a dynamical systems theory approach to the present results, RSNs can be conceived of as “attractors,” i.e., patterns of activity into which the brain tends to gravitate for short periods of time (Deco et al., 2009; Hellyer et al., 2014). A macro-state of consciousness (such as normal waking, deep sleep or the psychedelic state) may, therefore, be graphically represented as an “attractor landscape” in which the depth of “basins of attraction” (valleys in an otherwise flat 2D-plane) reflect the stability of particular RSNs or metastable “sub-states,” i.e., more long lasting sub-states will have deep basins of attraction and unstable sub-states will have shallow ones. A recent paper (Kanamaru et al., 2013) has described brain function in these terms, suggesting that the shape of attractors depends on selective attention. In this particular model, high levels of acetylcholine activating muscarinic receptors were found to produce an attractor landscape with more stable sub-states. Relating this to the present results, the increased RSFC observed between different RSNs could be interpreted as a flattening of the attractor landscape, in which the basins of attraction are shallower, implying that the global system will move more easily between different metastable sub-states. A flattened (but not flat) attractor landscape would be consistent with increased “information” in the sense of the “information-integration” theory of consciousness (Tononi, 2012) since greater movement between metastable sub-states would imply that a larger number of these sub-states (or a broader “repertoire”) can be entered over a given time. At a critical flatness, the size of the repertoire of metastable states will be maximal but if the landscape is too flat, information will be reduced because attractors will become too unstable. This scenario is referred to as “super-criticality” (Chialvo, 2010), and if taken to the extreme, an entirely flat landscape would imply that the system has no metastable states, or just one entirely disordered one. Future studies are required to determine whether the psychedelic state is “critical” or “super-critical” in this sense (Tagliazucchi et al., 2012; Carhart-Harris et al., 2014a). Another way these results could be perceived however, is that increased between-RSN RSFC under psilocybin is representative of a “sub-critical” system, i.e., one that is more globally synchronous and therefore ordered; however, that there were also decreases in between-RSN RSFC under psilocybin, does not support this view. We intend to follow-up this matter in order to test our hypothesis that it is specifically the *ease of transition* (or transition probability) between RSNs/metastable sub-states that is facilitated under the drug.

In contrast to the marked changes in between-network RSFC observed with psilocybin, only one RSN-pair showed a significant change in RSFC under MDMA, i.e., increased ECN-DMN2 RSFC (Figure 3F). This result is difficult to interpret in isolation; however, it is worth noting that ECN-DMN2 RSFC was also significantly increased under psilocybin (Figure 3C). MDMA is not considered a classic psychedelic, although like psilocybin, its subjective effects are known to be significantly mediated by serotonergic mechanisms (Liechti and Vollenweider, 2001; Van Wel et al., 2011). Thus, increased ECN-DMN2 RSFC may relate to a shared aspect of these drugs' subjective effects, such as their propensity to alter mood and cognition (Carhart-Harris et al., 2014b). Pre-treatment studies with selective receptor antagonists would help to inform these matters.

There is an important caveat to be addressed about the present analysis. It should be noted that the two studies from which the data was derived employed quite different methodologies (e.g., intravenous administration of psilocybin vs. oral administration of MDMA, different MR scanners and different study samples). Thus, it would be problematic to attempt to make inferences based entirely on a comparison of their relative RSFC profiles. This analysis was not intended to be a formal comparison of the brain effects of MDMA and psilocybin and if this was the intention, then a standardized methodology would need to be employed. Rather, the present analysis has focused on understanding the neural correlates of the psychedelic state as produced by the classic psychedelic, psilocybin, and the finding that MDMA had a less marked effects on between-network RSFC has merely served to emphasize that the psychedelic state rests on a particularly profound disturbance of brain function. This does not imply that MDMA's own subjective effects are unimportant or that they do not involve some (albeit more subtle) changes in between-network RSFC.

The significant change in head movement under psilocybin implies that some of the results should be interpreted with caution, in particular the decreases in coupling strength. We have used multiple ways to model motion as a possible confound but for a subset of the RSN pairs, the changes with drug correlate with the differences in mean motion. These significant correlations do not necessarily mean that motion is responsible for these changes, since intensity of drug is likely to be associated with increased movement, meaning that disambiguating the two effects is problematic for some RSN pairs. In support of this, we found a marginally significant correlation between changes in motion and changes in the subjective intensity rating (*r* = 0.382, *p* = 0.08). Future work restricting head motion in the scanner and with larger samples is necessary to be able to demonstrate that changes in these RSN pairs that correlate with motion reflect genuine brain activity or not.

In conclusion, this new analysis has used between-network functional connectivity to investigate the effects of two distinct serotonergic compounds on spontaneous brain function. It was found that psilocybin produced marked changes in between-network RSFC, generally in the direction of increased coupling between RSNs, with an additional decrease in coupling between visual and sensorimotor networks. MDMA had a notably less marked effect on between-network RSFC implying that psilocybin's more profound effects on global brain function (at least as determined by this measure) may explain its more profound effects on consciousness. The analytic methods used in this study, i.e., using ICA templates to determine functional connectivity matrixes for different drug states, may have wider application, enabling researchers to more objectively describe and potentially categorize different states of consciousness.

### Conflict of interest statement

The authors declare that the research was conducted in the absence of any commercial or financial relationships that could be construed as a potential conflict of interest.

## References

[B1] Beckmann C. F. Deluca M. Devlin J. T. Smith S. M. (2005). Investigations into resting-state connectivity using independent component analysis. Philos. Trans. R. Soc. B Biol. Sci. 360, 1001–1013 10.1098/rstb.2005.1634 16087444 PMC1854918

[B2] Beckmann C. F. Mackay C. E. Filippini N. Smith S. M. (2009). Group comparison of resting-state FMRI data using multi-subject ICA and dual regression. Neuroimage 47, S148 10.1016/S1053-8119(09)71511-3

[B3] Biswal B. Yetkin F. Z. Haughton V. M. Hyde J. S. (1995). Functional connectivity in the motor cortex of resting human brain using echo-planar MRI. Magn. Reson. Med. 34, 537–541 10.1002/mrm.1910340409 8524021

[B4] Carhart-Harris R. L. Erritzoe D. Williams T. Stone J. M. Reed L. J. Colasanti A. (2012a). Neural correlates of the psychedelic state as determined by fMRI studies with psilocybin. Proc. Natl. Acad. Sci. U.S.A. 109, 2138–2143 10.1073/pnas.1119598109 22308440 PMC3277566

[B5] Carhart-Harris R. L. Leech R. Erritzoe D. Williams T. M. Stone J. M. Evans J. (2012b). Functional connectivity measures after psilocybin inform a novel hypothesis of early psychosis. Schizophr. Bull. 39, 1343–1351 10.1093/schbul/sbs117 23044373 PMC3796071

[B6] Carhart-Harris R. L. Leech R. Tagliazucchi E. Hellyer P. J. Chialvo D. R. Feilding A. (2014a). The entropic brain: A theory of conscious states informed by neuroimaging research with psychedelic drugs. Front. Hum. Neurosci. 8:20 10.3389/fnhum.2014.00020 24550805 PMC3909994

[B7] Carhart-Harris R. L. Murphy K. Leech R. Erritzoe D. Wall M. B. Bart F. (2014b). The effects of acutely administered 3, 4-methylenedioxymethamphetamine on spontaneous brain function in healthy volunteers measured with arterial spin labelling and blood oxygen level-dependent resting-state functional connectivity. Biol. Psychiatry. 10.1016/j.biopsych.2013.12.015 [Epub ahead of print]. PMC4578244 24495461

[B8] Carhart-Harris R. L. Williams T. M. Sessa B. Tyacke R. J. Rich A. S. Feilding A. (2011). The administration of psilocybin to healthy, hallucinogen-experienced volunteers in a mock-functional magnetic resonance imaging environment: a preliminary investigation of tolerability. J. Psychopharmacol. 25, 1562–1567 10.1177/0269881110367445 20395317

[B9] Chialvo D. R. (2010). Emergent complex neural dynamics. Nat. Phys. 6, 744–750 10.1038/nphys1803

[B10] Damoiseaux J. S. Greicius M. D. (2009). Greater than the sum of its parts: a review of studies combining structural connectivity and resting-state functional connectivity. Brain Struct. Funct. 213, 525–533 10.1007/s00429-009-0208-6 19565262

[B11] De Araujo D. B. Ribeiro S. Cecchi G. A. Carvalho F. M. Sanchez T. A. Pinto J. P. (2012). Seeing with the eyes shut: neural basis of enhanced imagery following ayahuasca ingestion. Hum. Brain Mapp. 33, 2550–2560 10.1002/hbm.21381 21922603 PMC6870240

[B12] Deco G. Rolls E. T. Romo R. (2009). Stochastic dynamics as a principle of brain function. Prog. Neurobiol. 88, 1–16 10.1016/j.pneurobio.2009.01.006 19428958

[B13] Dovern A. Fink G. R. Fromme A. C. Wohlschlager A. M. Weiss P. H. Riedl V. (2012). Intrinsic network connectivity reflects consistency of synesthetic experiences. J. Neurosci. 32, 7614–7621 10.1523/JNEUROSCI.5401-11.2012 22649240 PMC6703581

[B14] Erritzoe D. Frokjaer V. G. Haahr M. T. Kalbitzer J. Svarer C. Holst K. K. (2010). Cerebral serotonin transporter binding is inversely related to body mass index. Neuroimage 52, 284–289 10.1016/j.neuroimage.2010.03.086 20382236

[B15] Fox M. D. Snyder A. Z. Vincent J. L. Corbetta M. Van Essen D. C. Raichle M. E. (2005). The human brain is intrinsically organized into dynamic, anticorrelated functional networks. Proc. Natl. Acad. Sci. U.S.A. 102, 9673–9678 10.1073/pnas.0504136102 15976020 PMC1157105

[B16] Friston K. J. Harrison L. Penny W. (2003). Dynamic causal modelling. Neuroimage 19, 1273–1302 10.1016/S1053-8119(03)00202-7 12948688

[B17] Gouzoulis-Mayfrank E. Hermle L. Kovar K. Sass H. (1996). Entactogenic drugs “ecstasy”(MDMA), “eve”(MDE) and other ring-substituted methamphetamine derivatives. A new class of substances among illegal designer drugs?]. Nervenarzt 67, 369 9005345

[B18] Green A. R. Mechan A. O. Elliott J. M. O'shea E. Colado M. I. (2003). The pharmacology and clinical pharmacology of 3, 4-methylenedioxymethamphetamine (MDMA,“ecstasy”). Pharmacol. Rev. 55, 463–508 10.1124/pr.55.3.3 12869661

[B19] Hellyer P. J. Shanahan M. Scott G. Wise R. J. Sharp D. Leech R. (2014). The control of global brain dynamics: opposing actions of frontoparietal control and default mode networks on attention. J. Neurosci. 34, 451–461 10.1523/JNEUROSCI.1853-13.2014 24403145 PMC3870930

[B20] Huxley A. (1959). The Doors of Perception and Heaven and Hell. Harmondsworth: Penguin Books

[B21] Jenkinson M. Bannister P. Brady M. Smith S. (2002). Improved optimization for the robust and accurate linear registration and motion correction of brain images. Neuroimage 17, 825–841 10.1006/nimg.2002.1132 12377157

[B22] Kanamaru T. Fujii H. Aihara K. (2013). Deformation of attractor landscape via cholinergic presynaptic modulations: a computational study using a phase neuron model. PLoS ONE 8:e53854 10.1371/journal.pone.0053854 23326520 PMC3543278

[B23] Kolbrich E. A. Goodwin R. S. Gorelick D. A. Hayes R. J. Stein E. A. Huestis M. A. (2008). Plasma pharmacokinetics of 3,4-methylenedioxymethamphetamine after controlled oral administration to young adults. Ther. Drug Monit. 30, 320–332 10.1097/FTD.0b013e3181684fa0 18520604 PMC2663855

[B24] Laureys S. (2005). The neural correlate of (un)awareness: lessons from the vegetative state. Trends Cogn. Sci. 9, 556–559 10.1016/j.tics.2005.10.010 16271507

[B25] Laureys S. Owen A. Schiff N. (2009). Coma science: clinical and ethical implications. Preface. Prog. Brain Res. 177, xiii–xiv 10.1016/S0079-6123(09)17736-1 19818889

[B26] Liechti M. E. Vollenweider F. X. (2001). Which neuroreceptors mediate the subjective effects of MDMA in humans? A summary of mechanistic studies. Hum. Psychopharmacol. 16, 589–598 10.1002/hup.348 12404538

[B27] Luke D. P. Terhune D. B. (2013). The induction of synaesthesia with chemical agents: a systematic review. Front. Psychol. 4:753 10.3389/fpsyg.2013.00753 24146659 PMC3797969

[B28] Mamah D. Barch D. M. Repovs G. (2013). Resting state functional connectivity of five neural networks in bipolar disorder and schizophrenia. J. Affect. Disord. 150, 601–609 10.1016/j.jad.2013.01.051 23489402 PMC3749249

[B29] Muthukumaraswamy S. D. Carhart-Harris R. L. Moran R. J. Brookes M. J. Williams T. M. Errtizoe D. (2013). Broadband cortical desynchronization underlies the human psychedelic state. J. Neurosci. 33, 15171–15183 10.1523/JNEUROSCI.2063-13.2013 24048847 PMC6618409

[B30] Nutt D. J. King L. A. Nichols D. E. (2013). Effects of Schedule I drug laws on neuroscience research and treatment innovation. Nat. Rev. Neurosci. 14, 577–585 10.1038/nrn3530 23756634

[B31] Power J. D. Barnes K. A. Snyder A. Z. Schlaggar B. L. Petersen S. E. (2012). Spurious but systematic correlations in functional connectivity MRI networks arise from subject motion. Neuroimage 59, 2142–2154 10.1016/j.neuroimage.2011.10.018 22019881 PMC3254728

[B32] Raichle M. E. Macleod A. M. Snyder A. Z. Powers W. J. Gusnard D. A. Shulman G. L. (2001). A default mode of brain function. Proc. Natl. Acad. Sci. U.S.A. 98, 676–682 10.1073/pnas.98.2.676 11209064 PMC14647

[B33] Riba J. Anderer P. Morte A. Urbano G. Jane F. Saletu B. (2002). Topographic pharmaco-EEG mapping of the effects of the South American psychoactive beverage ayahuasca in healthy volunteers. Br. J. Clin. Pharmacol. 53, 613–628 10.1046/j.1365-2125.2002.01609.x 12047486 PMC1874340

[B34] Satterthwaite T. D. Elliott M. A. Gerraty R. T. Ruparel K. Loughead J. Calkins M. E. (2013). An improved framework for confound regression and filtering for control of motion artifact in the preprocessing of resting-state functional connectivity data. Neuroimage 64, 240–256 10.1016/j.neuroimage.2012.08.052 22926292 PMC3811142

[B35] Shannon C. E. Weaver W. (1949). The Mathematical Theory of Communication. Urbana, IL: University of Illinois Press

[B36] Sinke C. Neufeld J. Emrich H. M. Dillo W. Bleich S. Zedler M. (2012). Inside a synesthete's head: a functional connectivity analysis with grapheme-color synesthetes. Neuropsychologia 50, 3363–3369 10.1016/j.neuropsychologia.2012.09.015 23000109

[B37] Smith S. M. (2002). Fast robust automated brain extraction. Hum. Brain Mapp. 17, 143–155 10.1002/hbm.10062 12391568 PMC6871816

[B38] Smith S. M. Brady J. M. (1997). SUSAN—a new approach to low level image processing. Int. J. Comput. Vis. 23, 45–78 10.1109/83.481674 18285093

[B39] Smith S. M. Fox P. T. Miller K. L. Glahn D. C. Fox P. M. Mackay C. E. (2009). Correspondence of the brain's functional architecture during activation and rest. Proce. Natl. Acad. Sci. U.S.A. 106, 13040–13045 10.1073/pnas.0905267106 19620724 PMC2722273

[B40] Smith S. M. Jenkinson M. Woolrich M. W. Beckmann C. F. Behrens T. E. Johansen-Berg H. (2004). Advances in functional and structural MR image analysis and implementation as FSL. Neuroimage 23, S208–S219 10.1016/j.neuroimage.2004.07.051 15501092

[B41] Tagliazucchi E. Balenzuela P. Fraiman D. Chialvo D. R. (2012). Criticality in large-scale brain FMRI dynamics unveiled by a novel point process analysis. Front. Physiol. 3:15 10.3389/fphys.2012.00015 22347863 PMC3274757

[B42] Tognoli E. Kelso J. (2014). The metastable brain. Neuron 81, 35–48 10.1016/j.neuron.2013.12.022 24411730 PMC3997258

[B43] Tononi G. (2004). An information integration theory of consciousness. BMC Neurosci. 5:42 10.1186/1471-2202-5-42 15522121 PMC543470

[B44] Tononi G. (2012). Integrated information theory of consciousness: an updated account. Arch. Ital. Biol. 150, 56–90 10.4449/aib.v149i5.1388 23165867

[B45] Van Den Heuvel M. Mandl R. Pol H. H. (2008). Normalized cut group clustering of resting-state FMRI data. PLoS ONE 3:e2001 10.1371/journal.pone.0002001 18431486 PMC2291558

[B46] Van Wel J. H. Kuypers K. P. Theunissen E. L. Bosker W. M. Bakker K. Ramaekers J. G. (2011). Blockade of 5-HT2 receptor selectively prevents MDMA-induced verbal memory impairment. Neuropsychopharmacology 36, 1932–1939 10.1038/npp.2011.80 21562484 PMC3154112

[B47] Vollenweider F. X. Leenders K. L. Scharfetter C. Maguire P. Stadelmann O. Angst J. (1997). Positron emission tomography and fluorodeoxyglucose studies of metabolic hyperfrontality and psychopathology in the psilocybin model of psychosis. Neuropsychopharmacology 16, 357–372 10.1016/S0893-133X(96)00246-1 9109107

[B48] Wise S. P. Boussaoud D. Johnson P. B. Caminiti R. (1997). Premotor and parietal cortex: corticocortical connectivity and combinatorial computations 1. Annu. Rev. Neurosci. 20, 25–42 10.1146/annurev.neuro.20.1.25 9056706

[B49] Wood J. Kim Y. Moghaddam B. (2012). Disruption of prefrontal cortex large scale neuronal activity by different classes of psychotomimetic drugs. J. Neurosci. 32, 3022–3031 10.1523/JNEUROSCI.6377-11.2012 22378875 PMC3531997

[B50] Yan C.-G. Cheung B. Kelly C. Colcombe S. Craddock R. C. Di Martino A. (2013). A comprehensive assessment of regional variation in the impact of head micromovements on functional connectomics. Neuroimage 76, 183–201 10.1016/j.neuroimage.2013.03.004 23499792 PMC3896129

