# Supplementary material for: The effects of psilocybin and MDMA on between-network resting state functional connectivity in healthy volunteers
Source: Front Hum Neurosci. 2014 May 27;8:204. doi: 10.3389/fnhum.2014.00204 (PMC4034428; doi:10.3389/fnhum.2014.00204)
Supplement: Supplementary file 1 [file DataSheet1.DOCX]

**Supplementary Material**

The effects of psilocybin and MDMA on between-network resting state functional connectivity in healthy volunteers

| Supplementary Table 1a. Mean β values for the placebo (psilocybin) condition | | | | | | | | | | | | | |
| --- | --- | --- | --- | --- | --- | --- | --- | --- | --- | --- | --- | --- | --- |
|  | 1 | 2 | 3 | 4 | 5 | 6 | 7 | 8 | 9 | 10 | 11 | 12 | 13 |
| 1 |  | 0.4831 | 0.4845 | 0.2514 | 0.3605 | 0.0329 | -0.1682 | 0.1123 | -0.0278 | 0.0696 | 0.243 | 0.2577 | -0.2033 |
| 2 | 0.4831 |  | 0.3863 | 0.2657 | 0.4263 | -0.2867 | -0.3211 | -0.249 | -0.1585 | -0.2363 | 0.1978 | -0.0192 | -0.0151 |
| 3 | 0.4845 | 0.3863 |  | 0.0667 | 0.1803 | -0.0478 | 0.1037 | 0.0783 | -0.043 | -0.0559 | -0.1065 | -0.1432 | 0.0824 |
| 4 | 0.2514 | 0.2657 | 0.0667 |  | 0.5663 | -0.392 | -0.1443 | 0.0902 | -0.0454 | -0.0632 | 0.3176 | -0.1629 | -0.1497 |
| 5 | 0.3605 | 0.4263 | 0.1803 | 0.5663 |  | -0.3076 | -0.2396 | -0.0999 | -0.1146 | -0.2883 | 0.2773 | -0.0331 | -0.102 |
| 6 | 0.0329 | -0.2867 | -0.0478 | -0.392 | -0.3076 |  | 0.4347 | 0.21 | -0.0156 | 0.1264 | -0.1765 | 0.1765 | -0.1285 |
| 7 | -0.1682 | -0.3211 | 0.1037 | -0.1443 | -0.2396 | 0.4347 |  | 0.3517 | 0.1577 | 0.1186 | -0.3257 | -0.2099 | -0.2131 |
| 8 | 0.1123 | -0.249 | 0.0783 | 0.0902 | -0.0999 | 0.21 | 0.3517 |  | 0.2404 | 0.4146 | 0.3026 | 0.2334 | -0.1925 |
| 9 | -0.0278 | -0.1585 | -0.043 | -0.0454 | -0.1146 | -0.0156 | 0.1577 | 0.2404 |  | 0.3071 | 0.0764 | 0.3957 | -0.1019 |
| 10 | 0.0696 | -0.2363 | -0.0559 | -0.0632 | -0.2883 | 0.1264 | 0.1186 | 0.4146 | 0.3071 |  | 0.2017 | 0.479 | -0.2317 |
| 11 | 0.243 | 0.1978 | -0.1065 | 0.3176 | 0.2773 | -0.1765 | -0.3257 | 0.3026 | 0.0764 | 0.2017 |  | 0.4036 | -0.1053 |
| 12 | 0.2577 | -0.0192 | -0.1432 | -0.1629 | -0.0331 | 0.1765 | -0.2099 | 0.2334 | 0.3957 | 0.479 | 0.4036 |  | -0.205 |
| 13 | -0.2033 | -0.0151 | 0.0824 | -0.1497 | -0.102 | -0.1285 | -0.2131 | -0.1925 | -0.1019 | -0.2317 | -0.1053 | -0.205 |  |

| Supplementary Table 1b. Mean β values for the psilocybin condition | | | | | | | | | | | | | |
| --- | --- | --- | --- | --- | --- | --- | --- | --- | --- | --- | --- | --- | --- |
|  | 1 | 2 | 3 | 4 | 5 | 6 | 7 | 8 | 9 | 10 | 11 | 12 | 13 |
| 1 |  | 0.6119 | 0.4416 | 0.0789 | 0.077 | 0.1751 | -0.1132 | 0.0318 | 0.2075 | 0.2542 | 0.3523 | 0.4964 | 0.02 |
| 2 | 0.6119 |  | 0.5108 | 0.1059 | 0.0915 | -0.0375 | -0.1954 | -0.1629 | 0.0719 | 0.0034 | 0.1845 | 0.2347 | 0.1275 |
| 3 | 0.4416 | 0.5108 |  | -0.1257 | -0.1178 | 0.0516 | 0.1689 | 0.1596 | 0.0958 | 0.0868 | -0.0069 | 0.0561 | 0.1522 |
| 4 | 0.0789 | 0.1059 | -0.1257 |  | 0.455 | -0.2211 | -0.034 | 0.2112 | 0.1201 | 0.1641 | 0.2622 | 0.0916 | -0.1893 |
| 5 | 0.077 | 0.0915 | -0.1178 | 0.455 |  | -0.2265 | -0.0776 | 0.0175 | 0.0145 | -0.0478 | 0.3256 | 0.1163 | -0.1398 |
| 6 | 0.1751 | -0.0375 | 0.0516 | -0.2211 | -0.2265 |  | 0.3122 | 0.1806 | 0.2415 | 0.2228 | 0.0994 | 0.3128 | -0.0171 |
| 7 | -0.1132 | -0.1954 | 0.1689 | -0.034 | -0.0776 | 0.3122 |  | 0.577 | 0.402 | 0.2347 | 0.0728 | 0.0346 | -0.1739 |
| 8 | 0.0318 | -0.1629 | 0.1596 | 0.2112 | 0.0175 | 0.1806 | 0.577 |  | 0.4689 | 0.5088 | 0.2941 | 0.2601 | -0.2366 |
| 9 | 0.2075 | 0.0719 | 0.0958 | 0.1201 | 0.0145 | 0.241 | 0.402 | 0.4689 |  | 0.3993 | 0.3288 | 0.4737 | -0.2065 |
| 10 | 0.2542 | 0.0034 | 0.0868 | 0.1641 | -0.0478 | 0.2228 | 0.2347 | 0.5088 | 0.3993 |  | 0.3618 | 0.572 | -0.3015 |
| 11 | 0.3523 | 0.1845 | -0.0069 | 0.2622 | 0.3256 | 0.0994 | 0.0728 | 0.2941 | 0.3288 | 0.3618 |  | 0.552 | -0.2394 |
| 12 | 0.4964 | 0.2347 | 0.0561 | 0.0916 | 0.1163 | 0.3128 | 0.0346 | 0.2601 | 0.4737 | 0.572 | 0.552 |  | -0.2249 |
| 13 | 0.02 | 0.1275 | 0.1522 | -0.1893 | -0.1398 | -0.0171 | -0.1739 | -0.2366 | -0.2065 | -0.3015 | -0.2394 | -0.2249 |  |

| Supplementary Table 1c. The difference between the psilocybin and placebo β value | | | | | | | | | | | | | |
| --- | --- | --- | --- | --- | --- | --- | --- | --- | --- | --- | --- | --- | --- |
|  | 1 | 2 | 3 | 4 | 5 | 6 | 7 | 8 | 9 | 10 | 11 | 12 | 13 |
| 1 |  | 1.8388 | -0.5103 | -1.8569 | *-2.4796 | 1.8413 | 0.8702 | -1.2765 | **5.2879 | **3.7212 | **2.751 | **5.1372 | **2.9386 |
| 2 | 1.8388 |  | 1.5778 | -1.7584 | **-3.320 | **3.3726 | 1.7709 | 1.4228 | **3.2705 | **3.5629 | -0.1799 | **2.798 | 1.786 |
| 3 | -0.51033 | 1.5778 |  | -1.9483 | **-2.767 | 1.0038 | 1.2863 | 0.99477 | 1.9717 | 1.6236 | 1.2422 | *2.4967 | 0.82368 |
| 4 | -1.8569 | -1.7584 | -1.9483 |  | -2.1843 | *2.4516 | 1.3543 | *2.3759 | **3.6057 | **5.5024 | -0.80522 | **4.4828 | -0.34738 |
| 5 | *-2.4796 | **-3.320 | **-2.77 | -2.1843 |  | 1.0305 | 2.1088 | **2.9525 | *2.5751 | **3.6544 | 0.84846 | *2.3498 | -0.50986 |
| 6 | 1.8413 | **3.3726 | 1.0038 | *2.4516 | 1.0305 |  | -1.5981 | -0.56799 | **3.6022 | 1.4368 | **3.2497 | 1.7488 | 1.0225 |
| 7 | 0.8702 | 1.7709 | 1.2863 | 1.3543 | 2.1088 | -1.5981 |  | **3.1482 | **2.9739 | 1.7997 | **4.4942 | **3.0226 | 0.53639 |
| 8 | -1.2765 | 1.4228 | 0.99477 | *2.3759 | **2.9525 | -0.56799 | **3.1482 |  | **3.1085 | **2.9851 | -0.10594 | 0.32027 | -0.52105 |
| 9 | **5.2879 | **3.2705 | 1.9717 | **3.6057 | *2.5751 | **3.6022 | **2.9739 | **3.1085 |  | 1.9053 | **3.6638 | 1.3049 | -1.6742 |
| 10 | **3.7212 | **3.5629 | 1.6236 | **5.5024 | **3.6544 | 1.4368 | 1.7997 | **2.9851 | 1.9053 |  | *2.6601 | 1.8836 | -1.4042 |
| 11 | **2.751 | -0.1799 | 1.2422 | -0.80522 | 0.84846 | **3.2497 | **4.4942 | -0.10594 | **3.6638 | *2.6601 |  | **2.7355 | -2.0438 |
| 12 | **5.1372 | **2.798 | *2.4967 | **4.4828 | *2.3498 | 1.7488 | **3.0226 | 0.32027 | 1.3049 | 1.8836 | **2.7355 |  | -0.22071 |
| 13 | **2.9386 | 1.786 | 0.82368 | -0.34738 | -0.50986 | 1.0225 | 0.53639 | -0.52105 | -1.6742 | -1.4042 | -2.0438 | -0.22071 |  |

**Supplementary Tables 1. Between-network resting state functional connectivity results in the psilocybin condition**. The three tables are: (a) Group mean of β values in the placebo condition. (b) Group mean of β values in the psilocybin condition. (c) Paired t test (2-tail) for the difference between the mean β values of each condition. The rows and columns represent the different RSNs. The networks from Smith et al. (2009) are: (1) Visual – Medial (VisM), (2) Visual – Lateral (VisL), (3) Visual – Occipital pole (VisO), (4) Auditory (AUD), (5) Sensorimotor (SM), (6) Default Mode Network (DMN), (7) DMN2 – A hybrid of anterior DMN and Executive Control Network, (8) Executive Control Network (ECN), (9) left Frontoparietal Network (lFP), (10) right Frontoparietal Network (rFP), (11) Dorsal Attention Network (DAN), (12) DAN2, (13) Cerebellum

FDR correction for multiple comparison (N = 78) was applied on the t-tests:

* 0.05 < q < 0.1

** q < 0.05

| Supplementary Table 2a. Mean β values for the placebo (MDMA) condition | | | | | | | | | | | | | |
| --- | --- | --- | --- | --- | --- | --- | --- | --- | --- | --- | --- | --- | --- |
|  | 1 | 2 | 3 | 4 | 5 | 6 | 7 | 8 | 9 | 10 | 11 | 12 | 13 |
| 1 |  | 0.4435 | 0.5705 | 0.1438 | 0.3547 | 0.1275 | -0.1829 | 0.0176 | -0.0021 | -0.0508 | 0.2157 | 0.2877 | 0.0305 |
| 2 | 0.4435 |  | 0.5169 | 0.3186 | 0.387 | -0.2154 | -0.2964 | -0.1383 | 0.0063 | -0.1295 | 0.333 | 0.1012 | 0.0059 |
| 3 | 0.5705 | 0.5169 |  | 0.1308 | 0.2776 | -0.08 | -0.1112 | 0.0466 | -0.0109 | -0.1205 | 0.0666 | 0.0619 | 0.0834 |
| 4 | 0.1438 | 0.3186 | 0.1308 |  | 0.4841 | -0.3961 | -0.0613 | 0.2153 | -0.0754 | 0.063 | 0.2157 | -0.2435 | 0.1033 |
| 5 | 0.3547 | 0.387 | 0.2776 | 0.4841 |  | -0.2361 | -0.2651 | 0.0337 | 0.0178 | -0.1106 | 0.4055 | 0.1381 | 0.0414 |
| 6 | 0.1275 | -0.2154 | -0.08 | -0.3961 | -0.2361 |  | 0.3669 | 0.0435 | 0.0961 | 0.0754 | -0.0769 | 0.2472 | -0.1349 |
| 7 | -0.1829 | -0.2964 | -0.1112 | -0.0613 | -0.2651 | 0.3669 |  | 0.1406 | 0.1541 | 0.1406 | -0.3037 | -0.2103 | -0.1713 |
| 8 | 0.0176 | -0.1383 | 0.0466 | 0.2153 | 0.0337 | 0.0435 | 0.1406 |  | 0.0982 | 0.2595 | 0.0925 | -0.0427 | 0.1092 |
| 9 | -0.0021 | 0.0063 | -0.0109 | -0.0754 | 0.0178 | 0.0961 | 0.1541 | 0.0982 |  | 0.2526 | 0.1182 | 0.3414 | 0.0145 |
| 10 | -0.0508 | -0.1295 | -0.1205 | 0.063 | -0.1106 | 0.0754 | 0.1406 | 0.2595 | 0.2526 |  | 0.0368 | 0.2494 | -0.0681 |
| 11 | 0.2157 | 0.333 | 0.0666 | 0.2157 | 0.4055 | -0.0769 | -0.3037 | 0.0925 | 0.1182 | 0.0368 |  | 0.3141 | -0.0245 |
| 12 | 0.2877 | 0.1012 | 0.0619 | -0.2435 | 0.1381 | 0.2472 | -0.2103 | -0.0427 | 0.3414 | 0.2494 | 0.3141 |  | -0.0264 |
| 13 | 0.0305 | 0.0059 | 0.0834 | 0.1033 | 0.0414 | -0.1349 | -0.1713 | 0.1092 | 0.0145 | -0.0681 | -0.0245 | -0.0264 |  |

| Supplementary Table 2b. Mean β values for the MDMA condition | | | | | | | | | | | | | |
| --- | --- | --- | --- | --- | --- | --- | --- | --- | --- | --- | --- | --- | --- |
|  | 1 | 2 | 3 | 4 | 5 | 6 | 7 | 8 | 9 | 10 | 11 | 12 | 13 |
| 1 |  | 0.5139 | 0.5871 | 0.2026 | 0.2561 | 0.1571 | -0.115 | 0.1018 | 0.0713 | 0.0167 | 0.2952 | 0.3415 | 0.0392 |
| 2 | 0.5139 |  | 0.4999 | 0.2654 | 0.2562 | -0.1782 | -0.2968 | -0.1238 | -0.0776 | -0.0597 | 0.3081 | 0.0291 | 0.0239 |
| 3 | 0.5871 | 0.4999 |  | 0.1006 | 0.1764 | -0.0489 | -0.0151 | 0.1234 | -0.0076 | -0.1317 | 0.1602 | 0.0908 | 0.0816 |
| 4 | 0.2026 | 0.2654 | 0.1006 |  | 0.4704 | -0.3992 | -0.1032 | 0.2813 | -0.0056 | 0.147 | 0.1752 | -0.2382 | 0.1254 |
| 5 | 0.2561 | 0.2562 | 0.1764 | 0.4704 |  | -0.1992 | -0.1756 | 0.0842 | -0.0073 | -0.0146 | 0.3725 | 0.1146 | 0.0362 |
| 6 | 0.1571 | -0.1782 | -0.0489 | -0.3992 | -0.1992 |  | 0.2738 | 0.0237 | 0.1106 | 0.0329 | -0.0160 | 0.2894 | -0.1249 |
| 7 | -0.115 | -0.2968 | -0.0151 | -0.1032 | -0.1756 | 0.2738 |  | 0.2701 | 0.2611 | 0.1163 | -0.3016 | -0.0874 | -0.0777 |
| 8 | 0.1018 | -0.1238 | 0.1234 | 0.2813 | 0.0842 | 0.0237 | 0.2701 |  | 0.1795 | 0.2808 | 0.035 | 0.0178 | -0.0351 |
| 9 | 0.0713 | -0.0776 | -0.0076 | -0.0056 | -0.0073 | 0.111 | 0.2611 | 0.1795 |  | 0.2142 | 0.0532 | 0.2835 | -0.1137 |
| 10 | 0.0167 | -0.0597 | -0.1317 | 0.147 | -0.0146 | 0.0329 | 0.1163 | 0.2808 | 0.2142 |  | 0.0623 | 0.2323 | -0.1837 |
| 11 | 0.2952 | 0.3081 | 0.1602 | 0.1752 | 0.3725 | -0.016 | -0.3016 | 0.035 | 0.0532 | 0.0623 |  | 0.3425 | -0.0515 |
| 12 | 0.3415 | 0.0291 | 0.0908 | -0.2382 | 0.1146 | 0.2894 | -0.0874 | 0.0178 | 0.2835 | 0.2323 | 0.3425 |  | -0.0612 |
| 13 | 0.0392 | 0.0239 | 0.0816 | 0.1254 | 0.0362 | -0.1249 | -0.0777 | -0.0351 | -0.1137 | -0.1837 | -0.0515 | -0.0612 |  |

**Supplementary Tables 2. Between-network resting state functional connectivity results in the MDMA condition**. The three tables are: (a) Group mean of β values in the placebo condition. (b) Group mean of β values in the MDMA condition. (c) Paired t test (2-tail) for the difference between the mean β values of each condition. The rows and columns represent the different RSNs. The networks from Smith et al. (2009) are: (1) Visual – Medial (VisM), (2) Visual – Lateral (VisL), (3) Visual – Occipital pole (VisO), (4) Auditory (AUD), (5) Sensorimotor (SM), (6) Default Mode Network (DMN), (7) DMN2 – A hybrid of anterior DMN and Executive Control Network, (8) Executive Control Network (ECN), (9) left Frontoparietal Network (lFP), (10) right Frontoparietal Network (rFP), (11) Dorsal Attention Network (DAN), (12) DAN2, (13) Cerebellum

FDR correction for multiple comparison (N = 78) was applied on the t-tests:

* 0.05 < q < 0.1

** q < 0.05

| Supplementary Table 2c. The difference between the MDMA and placebo β value | | | | | | | | | | | | | |
| --- | --- | --- | --- | --- | --- | --- | --- | --- | --- | --- | --- | --- | --- |
|  | 1 | 2 | 3 | 4 | 5 | 6 | 7 | 8 | 9 | 10 | 11 | 12 | 13 |
| 1 |  | 1.1213 | 0.42843 | 0.9631 | -2.3439 | 0.64654 | 1.6997 | 1.0707 | 1.9116 | 1.2338 | 2.0587 | 1.1915 | 0.17522 |
| 2 | 1.1213 |  | -0.26865 | -1.108 | -2.7934 | 0.70937 | -0.0069 | 0.2096 | -1.6636 | 1.2332 | -0.81757 | -1.2573 | 0.37119 |
| 3 | 0.42843 | -0.26865 |  | -0.48652 | -2.0375 | 0.61559 | 1.6325 | 0.98425 | 0.086009 | -0.29354 | 1.9622 | 0.61664 | -0.05175 |
| 4 | 0.9631 | -1.108 | -0.48652 |  | -0.28493 | -0.08153 | -0.6622 | 1.074 | 1.5497 | 1.882 | -0.64379 | 0.21674 | 0.35329 |
| 5 | -2.3439 | -2.7934 | -2.0375 | -0.28493 |  | 0.8113 | 1.514 | 0.92837 | -0.44821 | 2.818 | -0.78419 | -0.49185 | -0.10269 |
| 6 | 0.64654 | 0.70937 | 0.61559 | -0.08153 | 0.8113 |  | -2.8998 | -0.33443 | 0.39801 | -0.87691 | 1.5838 | 0.81389 | 0.31954 |
| 7 | 1.6997 | -0.00691 | 1.6325 | -0.66218 | 1.514 | -2.8998 |  | **5.4865 | 2.8904 | -1.2793 | 0.053074 | 1.9037 | 2.4028 |
| 8 | 1.0707 | 0.2096 | 0.98425 | 1.074 | 0.92837 | -0.33443 | **5.4865 |  | 2.3268 | 0.37255 | -1.3495 | 1.2858 | -3.3378 |
| 9 | 1.9116 | -1.6636 | 0.086009 | 1.5497 | -0.44821 | 0.398 | 2.8904 | 2.3268 |  | -0.78582 | -1.3507 | -1.7059 | -2.7831 |
| 10 | 1.2338 | 1.2332 | -0.29354 | 1.882 | 2.818 | -0.87691 | -1.2793 | 0.37255 | -0.78582 |  | 1.231 | -0.33312 | -3.0641 |
| 11 | 2.0587 | -0.81757 | 1.9622 | -0.64379 | -0.78419 | 1.5838 | 0.05307 | -1.3495 | -1.3507 | 1.231 |  | 0.67108 | -0.62638 |
| 12 | 1.1915 | -1.2573 | 0.61664 | 0.21674 | -0.49185 | 0.81389 | 1.9037 | 1.2858 | -1.7059 | -0.33312 | 0.67108 |  | -0.67275 |
| 13 | 0.17522 | 0.37119 | -0.05175 | 0.35329 | -0.10269 | 0.31954 | 2.4028 | -3.3378 | -2.7831 | -3.0641 | -0.62638 | -0.67275 |  |
